# Characteristics of Interaction Between Caregivers and Children with Chronic Diseases in Oral Medication-Taking Situations: A Validation Study of the Interaction Rating Scale

**DOI:** 10.1007/s10995-025-04099-2

**Published:** 2025-05-12

**Authors:** Takuya Yasumoto, Tomoka Yamamoto, Atsuko Ishii, Hiroko Okuno, Haruo Fujino

**Affiliations:** 1 United Graduate School of Child Development, The University of Osaka, 2-2 Yamadaoka, Suita, Osaka 5650871 Japan; 2 School of Nursing, Sugiyama Jogakuen University, Nagoya, Aichi Japan; 3 Molecular Research Center for Children’s Mental Development, United Graduate School of Child Development, The University of Osaka, Suita, Osaka Japan; 4 Graduate School of Nursing of Health and Human Science, Osaka Metropolitan University, Habikino, Osaka Japan; 5 Graduate School of Human Sciences, The University of Osaka, Suita, Osaka Japan

**Keywords:** Caregiver, Child, Interaction, Chronic disease, Medication

## Abstract

**Introduction:**

Caregiver–child interaction is essential for maintaining adaptive oral medication-taking behavior in children. To evaluate interactive behavior between children and caregivers, the Interaction Rating Scale (IRS), an observation-based instrument for evaluating the quality of caregiver–child interaction, can be applied via observation of interactions. This study examined the applicability of the IRS in oral medication-taking situations.

**Methods:**

Sixty-six caregiver-child dyads were evaluated using the IRS. The reliability of the measure was evaluated using Cronbach’s alpha for internal consistency and intra-class coefficient (ICC) for inter-rater reliability and test–retest reliability. The concurrent validity was evaluated using the Positive and Negative Parenting Scale and the Social Skills Scale for Preschool Children.

**Results:**

The IRS total, caregiver, and child scores showed high internal consistency (α = 0.86–0.92), test–retest reliability (ICC = 0.76–0.80) and inter-rater reliability (ICC = 0.86–0.91). The IRS indices were partially associated with the Positive and Negative Parenting Scale and Social Skills Scale scores in the hypothesized directions.

**Discussion:**

The results indicated the IRS is a reliable and validated instrument for measuring characteristics of caregiver–child interactions in medication-taking situations. Further studies may be helpful for validating the measure in wider patient groups and investigating the medication behavior of children.

**Supplementary Information:**

The online version contains supplementary material available at 10.1007/s10995-025-04099-2.

## Significance

Inappropriate medication behavior can lead to negative consequences for patients. Prior research has suggested that parental involvement is an essential factor in maintaining children's good medication-taking behaviors, while validated measures of the quality of caregiver–child interactions are not available. This study examined the applicability of the Interaction Rating Scale in medication situations as a measure of the quality of the interaction between children and caregivers. The finding supports the use of the measure for assessing caregiver–child interactions in medication-taking situations. A behavioral measure of caregiver–child interactions may help future research to develop good medication-taking behaviors in children.

**Supplementary Information:**

The online version contains supplementary material available at 10.1007/s10995-025-04099-2.

## Introduction

Caregiver–child interactions play an essential role in medication-taking behavior. Maintaining good medication behavior is important for patients with chronic diseases to control their conditions. As therapeutic efficacy is often related to drug dosage, inadequate medication behavior (e.g., forgetting or refusal) leads to negative outcomes for patients, including reduced treatment response, treatment failure, relapse, and increased healthcare costs (Aslani & Schneider, 2014; Brown et al., 2016; Chen et al., 2024; World Health Organization, 2003). Several previous reports have identified risk factors for inadequate oral medication behavior in children, such as characteristics related to physical function (e.g., immaturity of swallowing), cognitive development, socialization, and dependence on caregivers for self-care (Chardon et al., 2022; Iio et al., 2011; Nagae et al., 2015; World Health Organization, 2003). Medication adherence in pediatric populations is a challenging issue caused by various factors, including palatability, dosage forms, and child acceptance (Kapoor et al., 2024). A recent study reported that 85% of parents of young children (aged 0–5 years) and 47% of parents of older children (aged 6–17 years) experienced difficulties in drug administration due to palatability or swallowing issues (Herziger et al., 2022). They also suggested that ideal drug formulations were highly heterogeneous and may differ between children and caregivers. Because parental involvement is one of the most important supports for medication-taking in children (Bhatia et al., 2014; Both et al., 2021; Bouchard et al., 2022; Jia et al., 2023; Kraenbring et al., 2019; Tan et al., 2021), the quality of caregiver–child interactions may be an essential factor for improving children’s psychosocial outcomes and medication behaviors (Jozefiak et al., 2019). Thus, it is important to assess the nature of caregiver–child interactions, to help strengthen the support caregivers provide in medication-taking situations (Ransone et al., 2018; Thomas et al., 2017). However, previous studies have only assessed the presence or absence of these interactions, and the quality of caregiver–child interactions has not been evaluated using a validated measure in medication-taking settings.

The Interaction Rating Scale (IRS) is a reliable, validated behavioral measure of the quality of caregivers’ interactions with children, and of children’s social skills (Anme et al., 2010). The IRS targets interactions between children and their caregivers and sets up situations in which the child works toward goal achievement through the caregiver’s involvement. Interactions are assessed in a routine and naturalistic setting by trained raters (Anme, 2009). The IRS assesses caregiver–child interactions and social competence (Anme et al., 2010). Although the IRS was originally developed to assess caregiver–child interactions in families and childcare practices, it may be applicable for evaluating the quality of caregiver–child interactions in medication-taking situations. A validated measure in evaluating caregiver–child interactions would help understanding the nature of the interaction and planning interventions to support medication taking. However, the applicability of the IRS for examining caregiver–child interactions in medication-taking situations has not been investigated.

### Aims

The purpose of the current study was to apply the IRS in medication-taking situations and evaluate the reliability and validity of the instrument as a measure of the quality of interaction between children and caregivers.

## Materials and Methods

### Participants

Caregivers and children aged between 0 and 8 years with a pediatric chronic disease (Information Center for Specific Pediatric Chronic Diseases, 2022), requiring medication, were recruited from pediatric wards of Aichi Children’s Health and Medical Center, Fujita Health University Hospital and Nagoya City University Hospital. The data were collected from February to July 2023. We recruited the inpatients from the pediatric ward to observe medication-taking behaviors and interactions between children and their caregivers. Demographic and clinical information was retrieved from medical records and interviews or questionnaires were conducted among caregivers.

Before enrollment in this study, written informed consent was obtained from the caregivers. This study was conducted in accordance with the Declaration of Helsinki and with the approval of the Research Ethics Committee of Graduate School of Human Sciences, Osaka University (Approval No.: 22095), Aichi Children’s Health and Medical Center Ethics Committee (Approval No.: 2022094), Sugiyama Jogakuen University (Approval No.: 116), and Nagoya City University (Approval No.: 3013–3).

### Rationale for Sample Size Estimation

On the basis of a published guideline for scale development (Prinsen et al., 2018), we planned to collect at least 50 observations, and 20 observations for the estimation of test–retest and inter-rater reliability. The sample sizes were considered the minimum numbers required to obtain reliable estimation in the coefficients.

### Procedure

The observations were performed in a hospital room or at the bedside. The facial expressions, speech, and behavior of the children and their caregivers during medication administration were recorded, from the time the medication was prepared to the time the child finished taking the medication. Twenty of the participants were assessed twice by the same rater to evaluate the test–retest reliability.

Caregiver–child interactions in medication-taking situations were video-recorded. Based on the video recordings, the raters assessed the interaction between caregivers and children during medication-taking behavior using the IRS protocol (Anme et al., 2010).

### Interaction Rating Scale (IRS)

The IRS is a reliable, validated measure of the quality of caregivers’ interactions with children, and of children’s social competence (Anme et al., 2010). The IRS was developed as a tool for assessing interactions between children and their caregivers and has been shown to have concurrent validity with other validated assessment measures (Anme, 2009), including the Nursing Child Assessment Teaching Scale (Barnard et al., 1989). The IRS was developed for children aged 0–8 years and their caregivers.

The IRS evaluates child and caregiver domains consisted with 10 subdomains. The child’s assessment consists of five subdomains (five items each): (1) Autonomy, (2) Responsiveness, (3) Empathy, (4) Motor regulation, and (5) Emotional regulation. The assessment of caregivers consists of five subdomains (nine items each): (6) Respect for autonomy, (7) Respect for responsiveness, (8) Respect for empathy, (9) Respect for cognitive development, and (10) Respect for social–emotional development (Anme et al., 2010). In addition, the overall relationship is rated on a 5-point scale. Three major indices were used for analysis: the child domain score, the caregiver domain score, and the IRS total score. Higher scores indicate better quality in the interaction. The IRS uses tasks for children that are designed to be slightly more difficult and more likely to generate interaction than the tasks they commonly perform at their respective developmental ages. Although tasks in the IRS are typically set up using toys, in the current study, “taking medication” itself was set as the task.

In this study, we observed interactions between the child and their caregiver while taking medication. To apply the IRS in medication-taking situations, the terms “task,” “playing games,” and “episode” in the original items were modified to “taking medication,” and “task materials” was modified to “medications” (26 items in total). Three researchers reviewed these modifications and obtained the permission of the original author of the IRS.

The IRS rating was conducted by two raters (TYam and AI) who had no contact with the participating children or their caregivers. The raters received training from the IRS developer and were licensed to conduct the assessment. They rated the IRS independently to calculate the inter-rater reliability.

### Concurrent Measures

To examine concurrent validity, caregivers completed the Positive and Negative Parenting Scale (PNPS) and the Social Skill Scale for preschool children (SSS).

#### Positive and Negative Parenting Scale (PNPS)

The PNPS is a measure to evaluate parenting attitudes and behaviors of caregivers (Ito et al., 2014). Caregivers rated each item using a 4-point Likert scale (from “none” to “very often”). The instrument consists of two major factors (i.e., positive parenting and negative parenting) and six subscales. In this study, we used Positive parenting, Positive responsivity, Respect for will, and Negative parenting as concurrent measures. The scores were converted to T-scores (PNPS Development Team, 2018). Higher scores indicate stronger attitudes toward the parenting behaviors.

#### Social Skills Scale for Preschool Children (SSS)

The SSS is a 24-item measure to evaluate social skills among preschool children (Anme et al., 2013). The SSS consists of three factors: Cooperation, Self-control, and Assertion. Each item was evaluated on a scale from 0 (none) to 2 (always). Self-control is appropriate behavior that suppresses one’s own needs and demands, and Assertion represents the ability to explain and express oneself and one's intentions clearly. We used the Self-control and Assertion subscales as concurrent measures.

### Data Analysis

#### Reliability of the IRS

We calculated Cronbach’s alpha to assess the internal consistency of the measures. The inter-rater reliability of the two raters and test–retest reliability were examined using intraclass correlation coefficients (ICCs). The second observation was conducted within a week. ICCs higher than 0.7 were considered to indicate high reliability.

#### Concurrent Validity of the IRS

To examine concurrent validity of the IRS, correlations between IRS indices and concurrent measures (the PNPS and SSS subscales) were examined by calculating Spearman’s correlation coefficients. We expected that higher IRS caregiver domain score would be correlated with higher scores for PNPS positive parenting indices (Positive parenting, Positive responsiveness, and Respect for will) and lower scores for Negative parenting. In addition, we expected that higher IRS scores would be correlated with higher SSS scores. These analyses were planned prior to data collection.

As exploratory analyses, we explored the association between age and the IRS total because social ability in interaction is considered to develop with age. Characteristics of the indices depending on developmental stages would be helpful for interpreting the indices. In addition, we examined the associations between the IRS and patient’s resistance to medication, caregiver’s resistance to medication (i.e., caregivers themselves feel uncomfortable giving medication), and caregiver’s devices for giving medications (i.e., caregivers are making any efforts to help the child), because previous studies identified these factors as predictors of medication behavior (Both et al., 2021; Chardon et al., 2022; Nagae et al., 2015). Independent t-tests were performed to examine the group differences in IRS total score. IBM SPSS Statistics ver. 28 (IBM, Armonk, NY, USA) was used to analyze the data. The significance threshold was set at 0.05.

## Results

A total of 78 caregivers and children participated in this study. After excluding data from 12 observations because of technical issues in videorecording the interaction, the final sample of the study was 66 children and their caregivers. The age of the children ranged 1 month to 8 years. The average age of the caregivers was 35.2 years. Most caregivers were mothers (88%). The demographic and clinical characteristics of the participants are shown in Table 1.

**Table 1:** Participants’ characteristics-column: cross-sectional; test–retest (n = 66)

Children		
Gender		
Girl	n (%)	25 (37.9)
Boy	n (%)	41 (62.1)
Age, months	Mean (SD); Range	29.6 (27.0); 1–96
Age groups		
< 12 months	n (%)	27 (40.9)
12–35 months	n (%)	16 (24.2)
≥ 36 months	n (%)	23 (34.8)
Number of types of medications	Mean (SD); Range	3.5 (2.2); 1–9
Resistance to medication (child is resistant to medication)		
Yes	n (%)	31 (47.0)
No	n (%)	35 (53.0)
Disease group		
Cardiac anomaly^a^	n (%)	46 (69.7)
Other^b^	n (%)	20 (30.3)
Caregiver		
Age, years	Mean (SD); Range	35.2 (7.9); 20–65
Relationship with a child		
Mothers	n (%)	58 (87.9)
Other	n (%)	8 (12.1)
Resistance to giving medication (caregivers themselves feel uncomfortable with giving medication)		
Yes	n (%)	27 (40.9)
No	n (%)	39 (59.1)
Device to get children to take the medication (parents are making any efforts to help the child)		
Yes	n (%)	51 (77.3)
No	n (%)	15 (22.7)

^a^The category included atrioventricular septal defect and ventricular septal defect, patent ductus arteriosus, and single ventricle

^b^The category included epilepsy, Kawasaki disease, acute leukemia, asthma and liver failure

The most common disease in children was cardiac anomaly (70%, including atrioventricular septal defect, ventricular septal defect, patent ductus arteriosus, and single ventricle). In the case of overlapping diseases, the major diagnosis was determined on the basis of the therapies given. The patients took 3.5 different medications on average (SD = 2.2). The number of medications was counted; for example, if a patient was prescribed one Teprenone capsule, one Prednisolone tablet, and two Enalapril Maleate tablets, the number of medications was three. Most patients were prescribed powder medication, and only three patients were prescribed tablets. Thirty-one children (47%) showed resistance to taking medication (e.g., difficulty swallowing because of taste, smell, or dosage form). Twenty-seven caregivers (41%) felt uncomfortable giving medication to their children, and 51 (77%) attempted to help the child take medication.

### Characteristics of IRS in Medication Settings

**Fig. 1:**
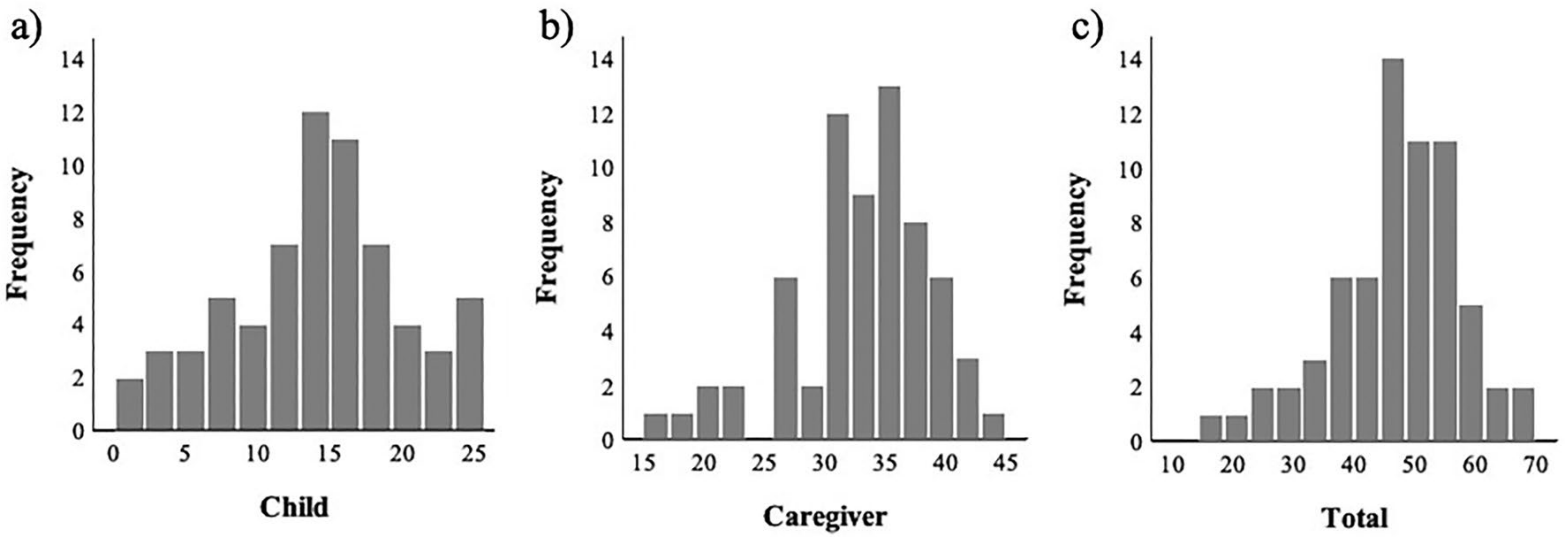
Distribution of the IRS scores (Child, Caregiver, Total). The three histograms show the distribution of each IRS score: **a** Child domain score, **b** caregiver domain score, and **c** total score. The x-axis shows the score of the indices and y-axis shows the frequency

**Fig. 2:**
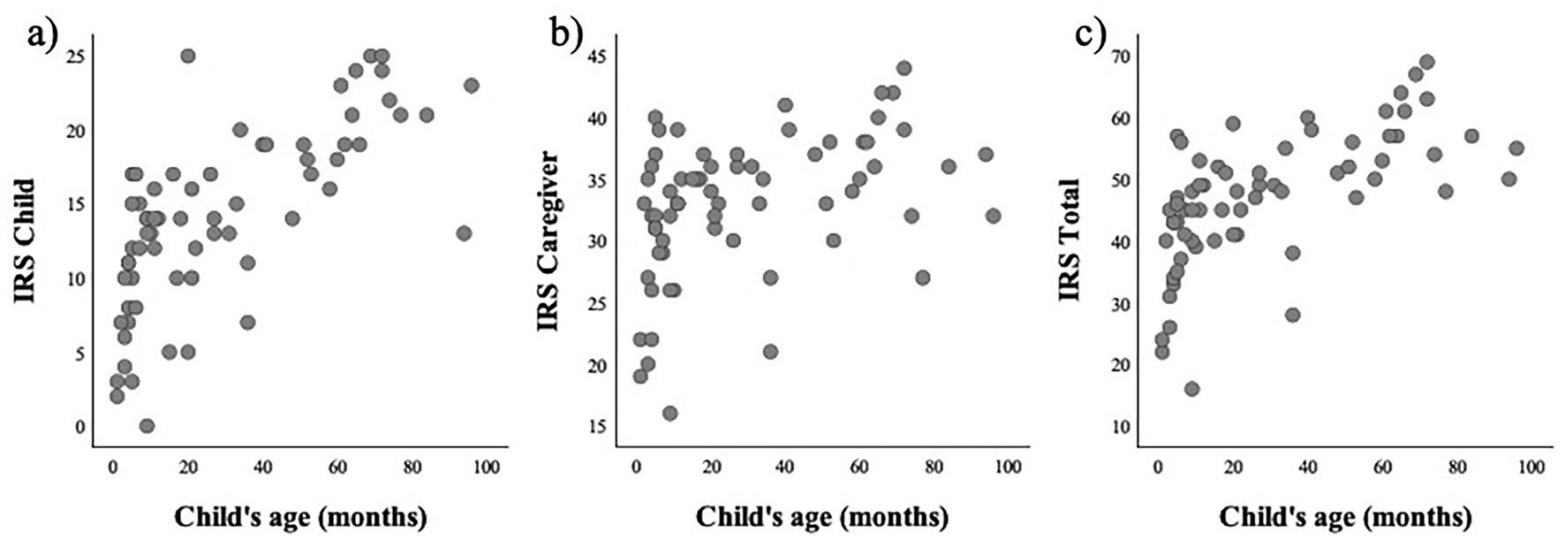
Correlation with child’s age and the IRS total score. The three scatter plots show the association between the child’s age and the IRS scores: **a** child domain score, **b** caregiver domain score, and **c** total score. The grey dots show individual data

Descriptive statistics for the IRS indices are shown in Table 2. The Cronbach’s alpha coefficient for the total scale was 0.92, and the coefficients for the child and caregiver domains were 0.91 and 0.86, respectively. The ICC for test–retest reliability was 0.80 for the total IRS score, 0.76 for the child domain, and 0.77 for the caregiver domain, indicating high test–retest reliability. Inter-rater reliability of the IRS scores was also high in the current sample (ICC = 0.86–0.91). The distributions of IRS total and caregiver domain scores in medication situations were not clearly different from the normal distribution (Fig. 1), while the child domain score indicated a weak ceiling effect. We examined the correlations between the age of the child and the IRS scores to explore the IRS score characteristics, which showed non-linear positive associations (Fig. 2).

**Table 2:** Descriptive statistics of the IRS (n = 66)

IRS	Mean (SD)	Min–max	Cronbach’s alpha	Test–retest reliability, ICC (95% CI), n = 20	Inter-rater reliability, ICC (95% CI), n = 17
Child	14.1 (6.1)	0–25	0.91	0.76 (0.49–0.90)	0.89 (0.72–0.96)
Caregiver	33.0 (5.8)	16–44	0.86	0.77 (0.51–0.90)	0.86 (0.66–0.95)
Total	47.1 (10.7)	16–69	0.92	0.80 (0.56–0.91)	0.91 (0.76–0.97)

*IRS* Interaction Rating Scale, *ICC* intraclass correlation coefficient, *CI* confidence interval

Based on the association between the IRS indices and child age, we further examined the reliability indices of the IRS in two age subgroups (children younger than 36 months and those 36 months or older). Cronbach’s alpha, test–retest reliability, and inter-rater reliability showed high or adequate reliability in most IRS indices, except for the child domain score in children younger than 36 months (ICC = 0.47; Supplementary Table S1). The ICC (test–retest reliability) of the child domain was affected by a few children who showed fluctuations between the two measurements, while the rest of the participants were within a certain range between the measurements (Supplementary Fig. S1).

Table 3 shows the correlations between each domain score of the IRS and the PNPS and SSS. There was a significant positive correlation between Positive response and the IRS caregiver and total scores (rho = .30, p = .022; rho = .32, p = .013). The two SSS indices showed positive correlations with the IRS scores (ps < .001). Unexpectedly, there was a significant negative correlation between the IRS child score and negative parenting (rho = 0.39, p = .002).

**Table 3:** Correlation between IRS and other concurrent measures (n = 66)

Concurrent measures	Descriptive statistics	Correlations with the IRS and concurrent measures		
		Child	Caregiver	Total
	Mean (SD)	rho	rho	rho
		[95%CI]	[95%CI]	[95%CI]
PNPS				
Positive parenting	50.3 (10.4)	0.13	0.14	0.16
		[− 0.14, 0.38]	[− 0.13, 0.39]	[− 0.11, 0.41]
Positive response	48.6 (9.5)	0.24	0.30*	0.32*
		[− 0.03, 0.47]	[0.04, 0.52]	[0.06, 0.54]
Respect for will	50.9 (10.4)	0.15	0.15	0.17
		[− 0.03, 0.47]	[− 0.12, 0.4]	[− 0.1, 0.42]
Negative parenting	48.3 (10.0)	0.39**	− 0.11	0.17
		[0.14, 0.59]	[− 0.37, 0.16]	[− 0.1, 0.42]
SSS				
Self-control	13.5 (5.2)	0.62**	0.52**	0.65**
		[0.42, 0.76]	[0.29, 0.69]	[0.47, 0.78]
Assertion	18.3 (3.9)	0.53**	0.44**	0.57**
		[0.31, 0.7]	[0.19, 0.63]	[0.35, 0.72]

*IRS* Interaction Rating Scale, *PNPS* Positive and Negative Parenting Scale, *SSS* Social Skills Scale for Preschool Children, *CI* Confidence Interval

**p < 0.01

*p < 0.05

### Exploratory Analysis

**Fig. 3:**
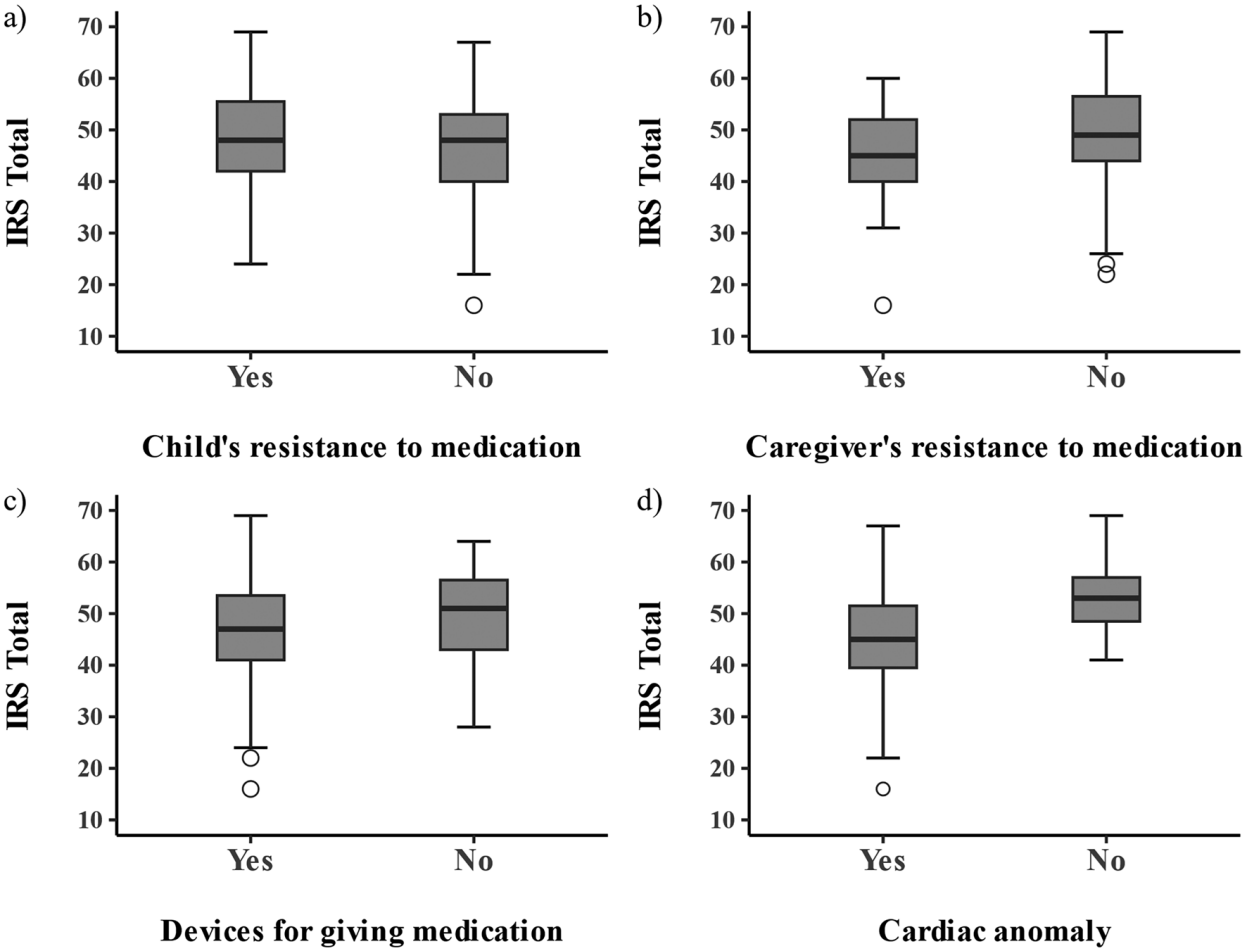
Group comparisons of the IRS scores. The boxplots show the comparisons of the IRS total score between the groups: **a** child’s resistance to medication, **b** caregiver’s resistance to medication, **c** devices for giving medication, and **d** Cardiac anomaly and other. There were no significant differences between the groups except the disease group (cardiac anomaly vs. other diseases, p < 0.001)

We compared the IRS total score using independent t-tests by the groups regarding the medication practice of the participants (Fig. 3). No significant associations were found between IRS scores and either child or caregiver resistance and IRS scores. Patients with cardiac anomaly had lower IRS total scores (p < 0.001), although the difference is expected to be affected by differences in age distribution (Supplementary Fig. S2).

## Discussion

The purpose of this study was to apply the IRS in medication-taking situations and evaluate the reliability and validity of the instrument as a measure of the quality of the interaction between children and caregivers. The results indicated good reliability and concurrent validity of the instrument in medication-taking situations, although further validation or refinement may be needed.

The psychometric properties of the measure were generally satisfactory (i.e., high internal consistency, inter-rater agreement, and test–retest reliability), despite our modifications to the wording of the scale items to apply the IRS in medication situations. These characteristics are comparable to the results of the original IRS validation study (Anme et al., 2010). However, the average scores and distributions of the IRS indices of this study differed from those of the original study (mean IRS total: 47.1 vs 62.2) (Anme et al., 2010). Such a difference may be explained by the age distribution of the current sample. The previous study reported IRS evaluations in children aged 18 and 30 months (Anme et al., 2010). Although the mean age in our sample (mean age = 29.6 months) was comparable to that in Anme et al.’s (2010) study, infants (< 12 months of age) comprised approximately 40% of the current study sample. Generally, vocal and physical interactive behaviors develop and increase in later age (Beuker et al., 2013; Mundy et al., 2007). The association between the child’s age and the IRS scores in the current study supported this notion (Fig. 2). Our additional examination of reliability in the two age subgroups suggested that the child domain may be susceptible to conditions or settings (e.g., such as mood, time, and environmental situations) in children younger than 36 months. While other domain scales showed adequate reliability indices, the measurement of the child domain in younger children requires careful interpretation. In addition to age, the task used for the IRS assessment (medication vs. playing a game) may also be a critical factor in determining the quality of interactions. Playing a game might be expected to facilitate social interactions between the children and caregivers, whereas medication-taking may have less of an interaction-promoting effect. Because the IRS evaluates caregiver–child interactions via observation, the context of the interaction may affect the nature of the interactions that occur.

On the basis of previous findings of a positive correlation between supportive parenting style and good medication behavior (Jia et al., 2023), we hypothesized the existence of a positive correlation between the PNPS and the IRS. The positive correlations with the IRS and SSS indices and Positive response supported the validity of the IRS, whereas other PNPS indices did not show significant correlations in the hypothesized directions. The lack of correlations between the IRS and PNPS indices may be partially explained by the age of the children. The social ability of children develops with age, and simultaneously, parental attitudes towards their children also change (Ito et al., 2014; PNPS Development Team, 2018). For example, negative parenting attitudes toward children may increase with age among the parents of preschoolers (Supplementary Fig. S3). Thus, associations should be interpreted in consideration of the age distribution of the sample. Another possible explanation is the discrepancy between self-report and behavioral observation in psychological constructs (Dang et al., 2020). For further validation, another type of behavioral observation or objective assessment may be required.

Many children have difficulty taking medications (Cain et al., 2020; Goode et al., 2003). Mothers may also have difficulty taking medication because of their children’s reluctance to take medication (Bouchard et al., 2022). Taken together, previous studies suggest that children’s resistance to medication and parental involvement in medication-taking situations are interrelated (Sonney et al., 2017; Tan et al., 2021). Contrary to expectations, the current results did not show differences in interactions between the groups based on the child’s resistance to medication, caregiver’s difficulty, and device for medication used by the caregiver. If these explanatory factors affected caregiver-child interactions, the differences may not have been sufficiently large to detect in the observation, or other characteristics of conditions of the patients, such as diseases and developmental age, may have affected the results. The current results showed a significant difference in IRS score by the disease group (i.e., cardiac anomaly and other). These findings may support those of a previous study indicating a higher level of distress in parents of children with cardiac anomaly (Golfenshtein et al., 2019); however, the difference in the age distribution of children in the study sample may not warrant a direct comparison between studies (Supplementary Fig. S2). The results should be interpreted cautiously.

## Limitation

The current study involved several limitations that should be considered. First, the current sample was characterized by children in infancy (41%) and children with cardiac anomaly (70%). Such characteristics may have affected the results of the current study. The inclusion of children with a wider range of diseases may strengthen the generalizability of the findings. Second, the participants in this study were recruited among inpatients at hospitals and observed under inpatient settings. Because child–caregiver interactions are likely to be affected by context and settings (e.g., at hospital vs. at home; taking medication vs. playing a game), further evaluation in consistent settings may be needed to confirm the association between parenting attitudes and behaviors in medication-taking. Third, the study did not adequately evaluate attitudes toward medication and behavior changes during medication-taking over the course of illness (Bhatia et al., 2014; Golfenshtein et al., 2019; Halling et al., 2021). Habitual medication behavior may decrease the motivation for interaction between a child and a caregiver. These characteristics may explain the lack of difference in the quality of interactions by resistance toward medication in children and caregivers.

## Implications for Practice

The current study suggests that the IRS can be applied as an instrument to assess the quality of child–caregiver interactions in medication settings in children with chronic diseases. Evaluating caregiver–child interaction is an essential aspect of assessing the factors influencing children’s medication-taking behavior (e.g., whether caregivers’ support leads to the maintenance of medication-taking behaviors). Previous studies showed that caregiver involvement, age of the child, cognitive factors, and social factors are essential in supporting medication-taking behaviors in pediatric patients (Cain et al., 2020; Sonney et al., 2017; Tan et al., 2021). Maintaining good medication-taking behaviors in children requires a multifaceted approach that includes direct support for children, as well as interventions for caregiver involvement (El-Rachidi et al., 2017). Interventions to support caregivers have been considered critical; however, detecting the effects of the interventions on parental interactive behaviors has been challenging. Detecting changes in interactive behaviors may be a key for understanding the factors that change medication-taking behaviors, and the IRS may provide useful information for future clinical practice and intervention research, although careful interpretation is required for the IRS child domain in younger children (< 36 months). The IRS may be a useful instrument for evaluating caregiver’s involvement from a behavioral perspective, and may provide a helpful tool in studies examining factors affecting children’s adherence to medication.

## Supplementary Information

Below is the link to the electronic supplementary material.

Supplementary file1 (PDF 363 KB)

## Data Availability

Due to the nature of this research, participants in this study did not agree for their data to be shared publicly, so supporting data are not available.
