# Correction: Characteristics of Interaction Between Caregivers and Children with Chronic Diseases in Oral Medication‑Taking Situations: A Validation Study of the Interaction Rating Scale

**DOI:** 10.1007/s10995-025-04180-w

**Published:** 2025-09-15

**Authors:** Takuya Yasumoto, Tomoka Yamamoto, Atsuko Ishii, Hiroko Okuno, Haruo Fujino

**Affiliations:** 1 United Graduate School of Child Development, The University of Osaka, 2‑2 Yamadaoka, Suita, Osaka 5650871 Japan; 2 School of Nursing, Sugiyama Jogakuen University, Nagoya, Aichi Japan; 3 Molecular Research Center for Children’s Mental Development, United Graduate School of Child Development, The University of Osaka, Suita, Osaka Japan; 4 Graduate School of Nursing of Health and Human Science, Osaka Metropolitan University, Habikino, Osaka Japan; 5 Graduate School of Human Sciences, The University of Osaka, Suita, Osaka Japan

**Correction to: Maternal and Child Health Journal (2025) 29:835–844**

10.1007/s10995-025-04099-2

The original version of this article unfortunately contained errors in affiliation and Results section. These errors do not affect the results, interpretations, or conclusions of the study.

In affiliations, the university name “Osaka University” should have been “The University of Osaka”.

The sentence “The age of the children ranged 5 months to 8 years.” should read as “The age of the children ranged 1 month to 8 years.”

The last sentence should read as “Twenty-seven caregivers (47%) felt uncomfortable giving medication to …. take medication.” should read as “Twenty-seven caregivers (41%) felt uncomfortable giving medication to …. take medication.”

In Table 1, the values 27 (47.0) and 39 (53.0) in the third column under "Resistance to giving medication (caregivers themselves feel uncomfortable with giving medication)" were incorrect and should have been 27 (40. 9) and 39 (59.1), respectively. For completeness and transparency, both old incorrect and correct version of the table is given here:

**Incorrect Table 1**.

**Table 1:** Participants’ characteristics-Column: Cross-sectional; Test-retest (*n* = 66)

Children		
Gender		
Girl	n (%)	25 (37.9)
Boy	n (%)	41 (62.1)
Age, months	Mean (SD); Range	29.6 (27.0); 1–96
Age groups		
< 12 months	n (%)	27 (40.9)
12–35 months	n (%)	16 (24.2)
≥ 36 months	n (%)	23 (34.8)
Number of types of medications	Mean (SD); Range	3.5 (2.2); 1–9
Resistance to medication (child is resistant to medication, )		
Yes	n (%)	31 (47.0)
No	n (%)	35 (53.0)
Disease group		
Cardiac anomaly^a^	n (%)	46 (69.7)
Other^b^	n (%)	20 (30.3)
Caregiver		
Age, years	Mean (SD); Range	35.2 (7.9); 20–65
Relationship with a child		
Mothers	n (%)	58(87.9)
Other	n (%)	8 (12.1)
Resistance to giving medication (caregivers themselves feel uncomfortable with giving medication),		
Yes	n (%)	27 (47.0)
No	n (%)	39 (53.0)
Device to get children to take the medication (parents are making any efforts to help the child)		
Yes	n (%)	51 (77.3)
No	n (%)	15 (22.7)

^a^The category included atrioventricular septal defect and ventricular septal defect, patent ductus arteriosus, and single ventricle

^b^The category included epilepsy, Kawasaki disease, acute leukemia, asthma and liver failure

**Corrected Table** **1**

**Table 1:** Participants’ characteristics-column: cross-sectional; test–retest (*n* = 66)

Children		
Gender		
Girl	n (%)	25 (37.9)
Boy	n (%)	41 (62.1)
Age, months	Mean (SD); Range	29.6 (27.0); 1–96
Age groups		
< 12 months	n (%)	27 (40.9)
12–35 months	n (%)	16 (24.2)
≥ 36 months	n (%)	23 (34.8)
Number of types of medications	Mean (SD); Range	3.5 (2.2); 1–9
Resistance to medication (child is resistant to medication, )		
Yes	n (%)	31 (47.0)
No	n (%)	35 (53.0)
Disease group		
Cardiac anomaly^a^	n (%)	46 (69.7)
Other^b^	n (%)	20 (30.3)
Caregiver		
Age, years	Mean (SD); Range	35.2 (7.9); 20–65
Relationship with a child		
Mothers	n (%)	58(87.9)
Other	n (%)	8 (12.1)
Resistance to giving medication (caregivers themselves feel uncomfortable with giving medication),		
Yes	n (%)	27 (40.9)
No	n (%)	39 (59.1)
Device to get children to take the medication (parents are making any efforts to help the child)		
Yes	n (%)	51 (77.3)
No	n (%)	15 (22.7)

^a^The category included atrioventricular septal defect and ventricular septal defect, patent ductus arteriosus, and single ventricle

^b^The category included epilepsy, Kawasaki disease, acute leukemia, asthma and liver failure

The original article has been corrected.

