# Supplementary material for: Characteristics of Interaction Between Caregivers and Children with Chronic Diseases in Oral Medication-Taking Situations: A Validation Study of the Interaction Rating Scale
Source: Matern Child Health J. 2025 May 12;29(6):835–44. doi: 10.1007/s10995-025-04099-2 (PMC12206201; doi:10.1007/s10995-025-04099-2)
Supplement: Supplementary file 1 — Supplementary file1 (PDF 363 KB) [file 10995_2025_4099_MOESM1_ESM.pdf]

Supplemental Information

Characteristics of Interaction Between Caregivers and Children with Chronic Diseases in Oral Medication-Taking Situations: A Validation Study of the Interaction Rating Scale

Takuya Yasumoto[a,b], Tomoka Yamamoto[a], Atsuko Ishii[c], Hiroko Okuno[d], Haruo Fujino[a,e]

a United Graduate School of Child Development, Osaka University, Suita, Osaka, Japan

b School of Nursing, Sugiyama Jogakuen University, Nagoya, Aichi, Japan

c Molecular Research Center for Children's Mental Development, United Graduate School of Child Development, Osaka University, Suita, Osaka, Japan

d Graduate School of Nursing of Health and Human Science, Osaka Metropolitan University, Habikino, Osaka, Japan

e Graduate School of Human Sciences, Osaka University, Suita, Osaka, Japan

Maternal and Child Health Journal

doi: 10.1007/s10995-025-04099-2

Supplemental Table S1. Descriptive statistics of the IRS of the subgroups divided by age group (36 months)

	Age < 36 months (n = 43)			Age ≥ 36 months (n = 23)		
IRS	Cronbach's alpha	Test-retest reliability, ICC (95% CI), n = 14	Inter-rater reliability, ICC (95% CI), n = 12	Cronbach's alpha	Test-retest reliability, ICC (95% CI), n = 6	Inter-rater reliability, ICC (95% CI), n= 5
Child	0.86	0.47 (-0.04-0.79)	0.78 (0.39-0.93)	0.88	0.86 (0.37-0.98)	-[a]
Caregiver	0.85	0.72 (0.34-0.90)	0.76 (0.37-0.93)	0.86	0.88 (0.45-0.98)	0.95 (0.53-0.99)
Total	0.88	0.67 (0.27-0.88)	0.80 (0.43-0.94)	0.91	0.91 (0.55-0.99)	0.99 (0.82-0.99)

[a] The ICC value of this cell were not computed due to an exact match.
IRS: Interaction Rating Scale
ICC: Intraclass correlation coefficient
CI: Confidence Interval

Supplemental Figure S1 top

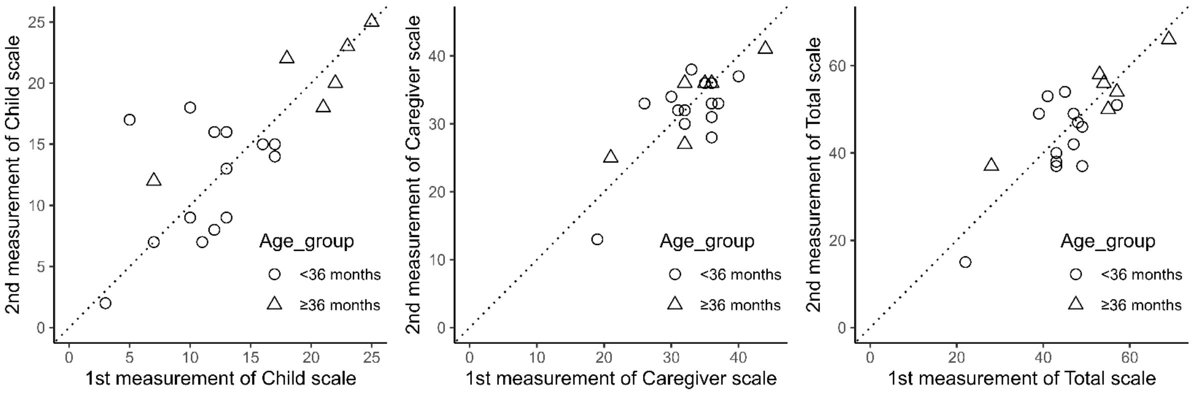

Supplemental Figure S1

Consistency between first and second measurements of the Interaction Rating Scale

Circles show data from children under 36 months of age. Triangles show data from children aged 36 months or older. The dot line is the reference line (y = x).

Supplemental Figure S2 top

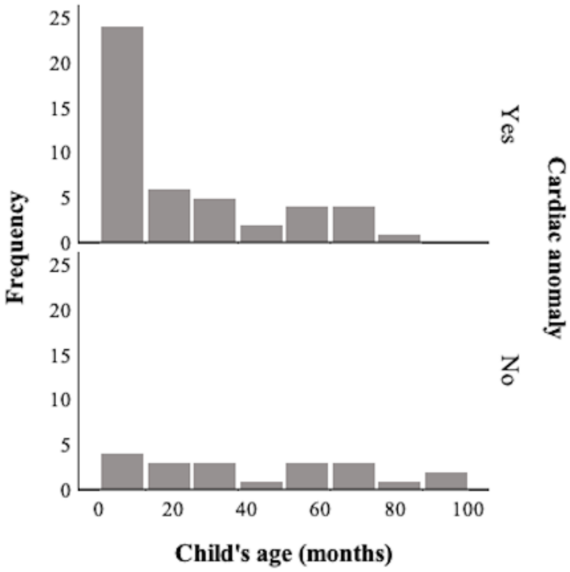

Supplemental Figure S2. **Distribution of the age in children with and without cardiac anomaly**

This histogram shows the respective frequencies in each group.

Supplemental Figure S3 top

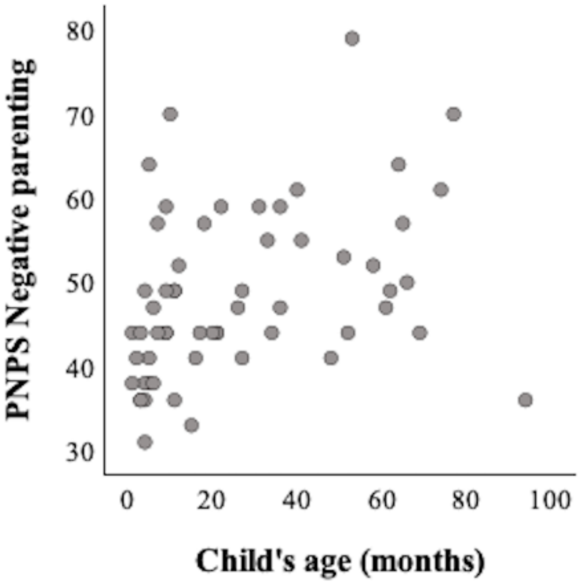

Supplemental Figure S3. Correlation with child's age and the PNPS Negative parenting score
